# 

**Published:** 1865-02

**Authors:** 


### BOOKS RECEIVED.

*Report on the condition of the Insane Poor in the County Poor Houses of New York.* BY SYLVESTER D. WILLARD, M. D.

*The Ophthalmic Review; a Quarterly Journal of Ophthalmic Surgery and Science. Edited by* J. ZACHARIAH LAURENCE, *of London, and* THOMAS WINDSOR, *of Manchester.*

*Fourteenth Anniversary Meeting of the Illinois State Medical Society, held in Chicago, May* 3, 4 *and* 5, 1864.

*Transactions of the Medical Society of the State of Pennsylvania, at its Fifteenth Annual Session, held in Philadelphia, June,* 1864.

*Defective and Impaired Vision, with the Clinical use of the Ophthalmoscope in their Diagnosis and Treatment.* BY LAURENCE TURNBULL, M. D., *Ophthalmic Surgeon to Howard Hospital; Member of the American Medical Association, etc. Philadelphia:* LINDSAY & BLAKISTON.

*Annual Returns of the Hamilton City Hospital; prepared by* DOCTOR STRANGE, *of the City Hospital.*

